# Comparative photochemistry activity and antioxidant responses in male and female *Populus cathayana* cuttings inoculated with arbuscular mycorrhizal fungi under salt

**DOI:** 10.1038/srep37663

**Published:** 2016-11-29

**Authors:** Na Wu, Zhen Li, Fei Wu, Ming Tang

**Affiliations:** 1 College of Life Sciences, Northwest A&F University, Yangling, 712100, Shaanxi, China; 2 College of Forestry, Northwest A&F University, Yangling, 712100, Shaanxi, China

## Abstract

We investigated the impact of arbuscular mycorrhizal fungi (AMF) on the morphology and physiology of two genders of the typical dioecious plant *Populus cathayana* under salt stress. We conducted a pot experiment containing seedlings of the two genders that were subjected to salt or non-salt and filled with soil that was either inoculated with *Rhizophagus intraradices* or not. The results showed that males had higher mycorrhizal dependency than females. Salt stress decreased growth, the relative water content and chlorophyll fluorescence. Meanwhile, salt increased the superoxide radical (O_2_^−^), and hydrogen peroxide (H_2_O_2_) contents and antioxidant enzyme activities. Mycorrhizal male seedlings performed better than females in shoot morphological growth under both conditions and in chlorophyll fluorescence parameters, O_2_^−^ and H_2_O_2_ contents, MDA concentration, proline content and antioxidant enzymes activities under salt stress. In females, under saline conditions, a lower MDA concentration and H_2_O_2_, O_2_^−^ and proline contents were observed in the leaves and roots. In addition, inoculated female plants performed better in chlorophyll fluorescence parameters than non-inoculated plants. AMF inoculation had either slight or no effects on the performance of females. These findings suggested that when subjected to stress and AMF, differences in the genders existed, followed by the alleviation of the damage to *P*. *cathayana* by AMF via improving growth and photosynthesis and antioxidant systems under salt stress.

Afforestation is a widely accepted solution for the remaining problems in the restoration of desert vegetation^1^. In addition, forests remain one of the most essential sources for energy and timber production^2^. As one important part, poplars are widespread in many areas and have been thoroughly studied as a model for elucidating specific mechanisms for trees^3^. Apart from their role in its experiment, poplars also play a key role in terrestrial ecosystems, especially under various kinds of abiotic stresses^3^,^4^,^5^.

The dioecious plant *Populus cathayana* Rehd., a native species in China, is considered to be an important reforestation species and is usually known as one of the most salt-sensitive woody plants in northwest China, one typically eco-fragile region^4^,^6^. Specific strategies of the two genders, especially in response to different kinds of stresses, are well-studied, and differences in their responses have been shown to be a result of different reproduction costs^4^. Different responses have been broadly detected in dioecious species between plants that bear male flowers and plants that bear female flowers^5^.

Large losses in arable land due to soil salinization are becoming a serious limitation to the expansion of agriculture and forestry around the world^7^. In addition to natural soil deposition processes, the use of irrigation water and fertilizers also contributes significantly to soil salinity^8^. Excessive salts limit the soil water available for seedlings, reduce seedling metabolism and nutrient uptake and result in osmotic imbalance. All these impacts contribute to stunted growth and production losses. Plants have various strategies to adapt to or avoid the damages caused by the activation of reactive oxygen species (ROS) and the accumulation of osmotic regulators^8^,^9^. That ROS generation and its scavenging system have a key effect in the defense of plants against various stresses has been well-documented^10^,^11^. As one typical abiotic stress, salt stress disrupts cellular metabolic homeostasis and promotes the production of ROS such as hydrogen peroxide (H_2_O_2_) and superoxide (O_2_^−^)^12^, and such ROS can damage plant organelles, inhibit photosynthesis and photo-chemical processes, and disturb ion homeostasis, eventually inducing the peroxidation of membrane lipids, the denaturation of proteins and the mutagenesis of DNA^13^. One of the most damaging oxidative effects is the peroxidation of membrane lipids, which results in the concomitant production of malondialdehyde (MDA)^12^. To facilitate the rapid removal of these compounds, plants possess antioxidant defense systems consisting of antioxidant enzymes including superoxide dismutase (SOD), peroxidase (POD), catalase (CAT) and the non-enzymatic antioxidant proline^14^. SOD is a key player in the antioxidant defense system for regulating the cellular concentration of O_2_^−^, which can catalyze the conversion of the superoxide anion into H_2_O_2_. H_2_O_2_ scavenging in the cellular machinery is very complex, mainly involving two antioxidant enzymes CAT and POD^15^. Thus, the ability to maintain a low level of ROS within plants is particularly important^16^.

Arbuscular mycorrhizal fungi (AMF) are ubiquitous fungi in the rhizosphere and are considered to be a novel biotechnological tools for enhancing salt resistance in plants in numerous ways^17^. AMF are integral components of all natural ecosystems and are reported to widely occur in saline soil. AMF alleviate salt stress by several possible mechanisms, including limiting ionic uptake, maintaining higher antioxidant enzymatic activities, improving nutrient uptake and providing a higher accumulation of osmosolutes^17^,^18^. In addition, Estrada *et al*.^18^ found that the effect of AMF symbiosis in inducing the activities of several antioxidant enzymes may be the indirect result of the mycorrhizal effects on host plant growth and photochemical activity. Evelin and Kapoor^19^ also found that AM symbiosis could improve antioxidative defense systems, facilitating the rapid dismutation of O_2_^−^ to H_2_O_2_, and the subsequent prevention of the production of H_2_O_2_ at higher intensities of salt stress. However, the response of individual enzymes has been shown to vary with respect to fungal species and the host plant. Hence, elucidating the mechanisms that control the enhanced antioxidant capacity in mycorrhizal plants under salt stress could provide a potential strategy to improve the tolerance of woody species.

AMF and dioecious plants are widely distributed in all terrestrial ecosystems. Although there have been some studies focusing on the functions of AMF in dioecious plants^20^,^21^,^22^,^23^, more systematic and wider researches are really needed. To investigate the impact of AMF and salt on male and female *P*. *cathayana* seedlings, we conducted the following experiment and hypothesized that: I) gender differences will exist when the seedlings are subjected to salt stress and AMF inoculation; II) salt stress will enhance antioxidant defense systems and limit plant growth and photochemical activity; III) salt tolerance can be enhanced in *P*. *cathayana* cuttings inoculated with AMF. In addition, we predicted that a gender imbalance would exist in dioecious plants in their adaptive strategies to abiotic stress and aimed to investigate whether AMF can reduce this imbalance.

## Results

### AM inoculation and mycorrhizal dependency

**Figure 1:**
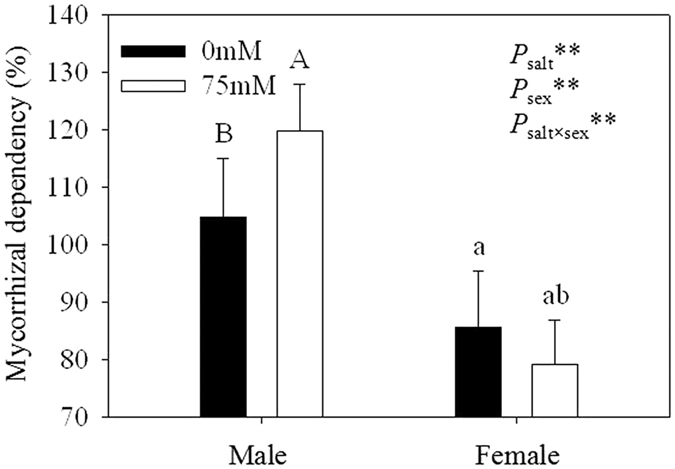
The mycorrhizal dependency of inoculated *P*. *cathayana* males and females under different salt conditions. Abbreviations: +M: AMF inoculation; −M: non-inoculation; 0 mM: without salt stress; 75 mM: under salt stress. Note: **: significant effect at *P* ≤ 0.01. Different letters (capital letters for males and lowercases for females) indicate significant difference at *p* ≤ 0.05.

Both genders in pots inoculated with *R. intraradices* formed typical AMF structures, while cuttings grown in pots that were not inoculated did not form mycorrhizal structures, and the roots were further confirmed with nested-PCR (data not shown)^24^. The colonization rate in all treatments was over 88%, and no significant differences existed in the AMF colonization rate between the genders or salt treatments. However, in the inoculated treatments, males showed higher mycorrhizal dependency than females under the same conditions (Fig. 1, Table 1). In addition, salt stress decreased the mycorrhizal dependency. Two-way ANOVAs illustrated that mycorrhizal dependency was significantly affected by salt, sex and the interaction of salt × sex.

### Growth measurement

**Figure 2:**
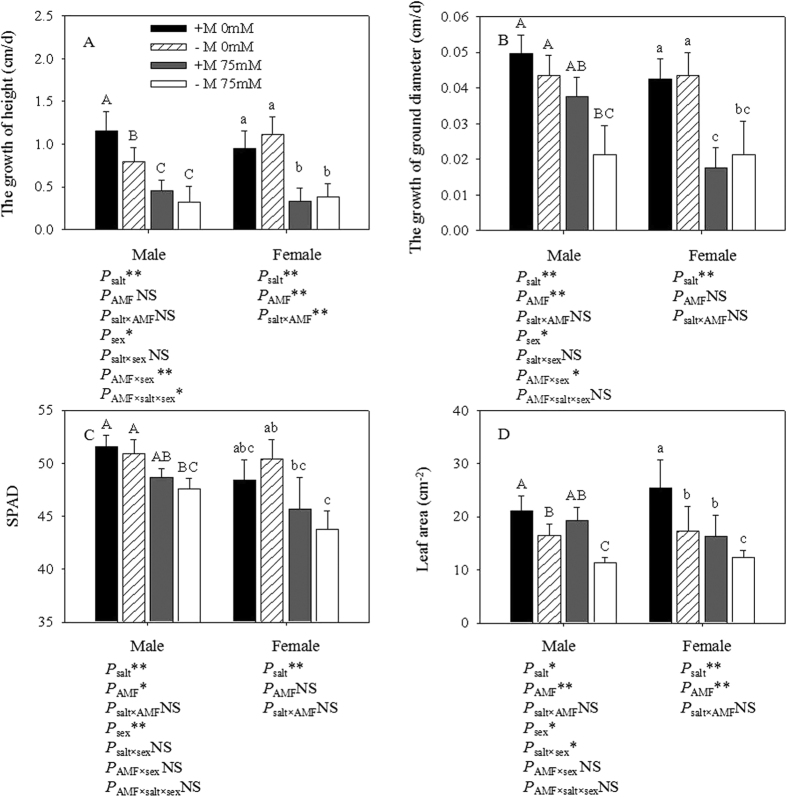
Effects of AMF inoculation on growth parameters of *P*. *cathayana* males and females under different salt conditions. Abbreviations: +M: AMF inoculation; −M: non-inoculation; 0 mM: without salt stress; 75 mM: under salt stress; AMF: AMF formation. Note: *: significant effect at 0.01 ≤ *P* ≤ 0.05; **: significant effect at *P* ≤ 0.01; NS: no significant effect. Different letters (capital letters for males and lowercases for females) indicate significant difference at p ≤ 0.05.

Females and males subjected to salt stress showed declines in growth in height (GH), growth in ground diameter (GGD), SPAD and leaf area (LA) (Fig. 2). Both genders grown without salt showed similar GGD and LA, but the GH and SPAD were significantly higher in males compared to females. Male and female cuttings differed in their response to AMF inoculation. For males, the GH, GGD, SPAD and LA of plants inoculated with AMF were 39.85%, 44.06%, 6.72% and 20.83% higher, respectively, than those of non-inoculated cuttings under saline conditions, whereas they were 14.29%, 7.38% lower and 4.32%, 19.02% higher, respectively, in females. Two-way ANOVAs showed that all the growth diameters measured in this study were significantly influenced by AMF, GH was significantly affected by salt, and LA in females was significantly affected by AMF. Three-way ANOVAs illustrated that GH was significantly affected by AMF and that GGD was significantly affected by sex.

### RWC measurement

**Figure 3:**
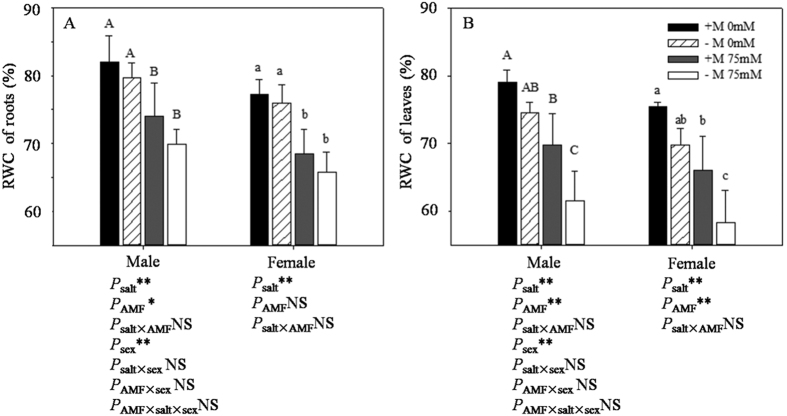
Effects of AMF inoculation on RWC of *P. cathayana* males and females roots (**A**) and leaves (**B**) under different salt conditions. Abbreviations: RWC: Relative water content; +M: AMF inoculation; −M: non-inoculation; 0 mM: without salt stress; 75 mM: under salt stress; AMF: AMF formation. Note: *: significant effect at 0.01 ≤ *P* ≤ 0.05; **: significant effect at *P* ≤ 0.01; NS: no significant effect. Different letters (capital letters for males and lowercases for females) indicate significant difference at p ≤ 0.05.

The RWC results are shown in Fig. 3. We compared the RWC of roots32 and shoots. We can see that, in the same treatment, roots showed a higher RWC than leaves, which was attributed to water transport from the roots to shoots. In addition, inoculated plants showed a higher RWC than seedlings without AMF in the same treatment, especially under salt stress. Salt significantly limited the RWC of both the roots and leaves. Two-way ANOVAs indicated that the proline content in both roots and leaves was significantly affected by salt and AMF inocula (except the RWC of female roots).

### Chlorophyll fluorescence measurements

**Figure 4:**
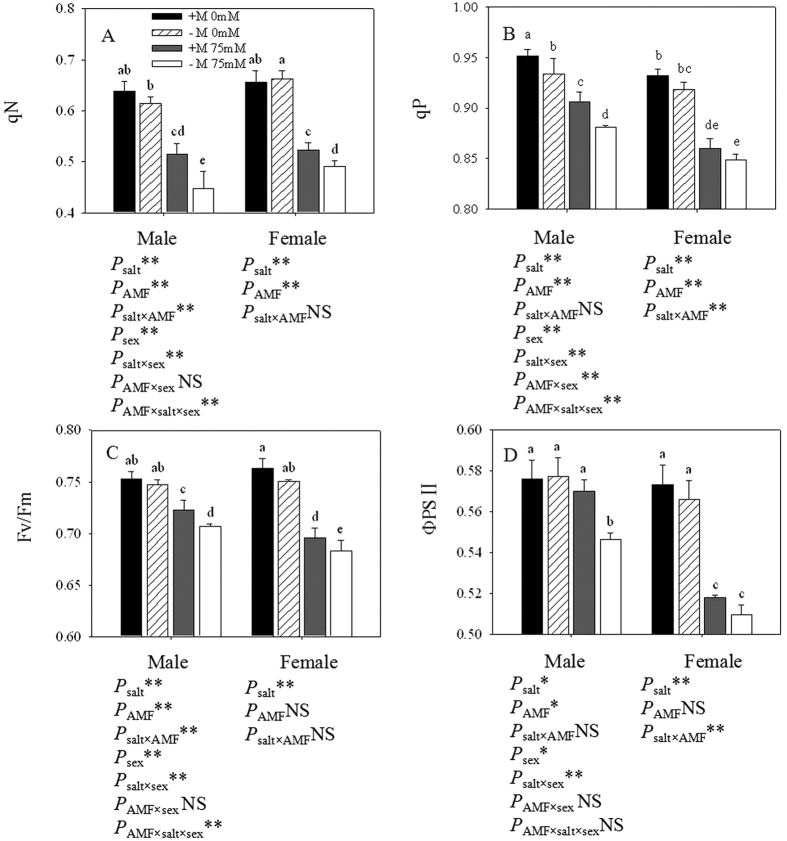
Effects of AMF inoculation on qN (**A**), qP (**B**), Fv/Fm (**C**) and ΦPSII (**D**) of *P*. *cathayana* males and females under different salt conditions. Abbreviations: qN: non-photochemical quenching coefficient; qP: photochemical quenching coefficient; Fv/Fm: maximal photosystem II quantum yield; ΦPSII: the efficiency of photosystem II; +M: AMF inoculation; −M: non-inoculation; 0 mM: without salt stress; 75 mM: under salt stress; AMF: AMF formation. Note: *: significant effect at 0.01 ≤ *P* ≤ 0.05; **: significant effect at *P* ≤ 0.01; NS: no significant effect. Different letters (capital letters for males and lowercases for females) indicate significant difference at p ≤ 0.05.

Salt stress significantly decreased the qN and qP in both sexes, especially in non-inoculated females (Fig. 4). Meanwhile, when exposed to salt stress, males exhibited a higher qP, Fv/Fm and ΦPSII than females. AMF inoculation showed positive effects on the qN, qP, Fv/Fm and ΦPSII in both sexes under salt stress. Under salt stress, compared with non-inoculated seedlings, inoculated male seedlings showed a higher qN (15.19%), qP (2.87%), Fv/Fm (2.25%), ΦPSII (4.30%), while inoculated female seedlings had a higher qN (6.75%), qP (1.37%), Fv/Fm (1.83%), and ΦPSII (1.65%). As we can see, inoculation had more positive effects on males than females, and on qN than the other chlorophyll fluorescence parameters. Two-way ANOVAs indicated that qN and Fv/Fm were significantly affected by salt × AMF in males, while qP and ΦPSII were significantly affected by salt × AMF in females. Three-way ANOVAs indicated that the four indexes were significantly affected by salt × sex.

### MDA measurement

**Figure 5:**
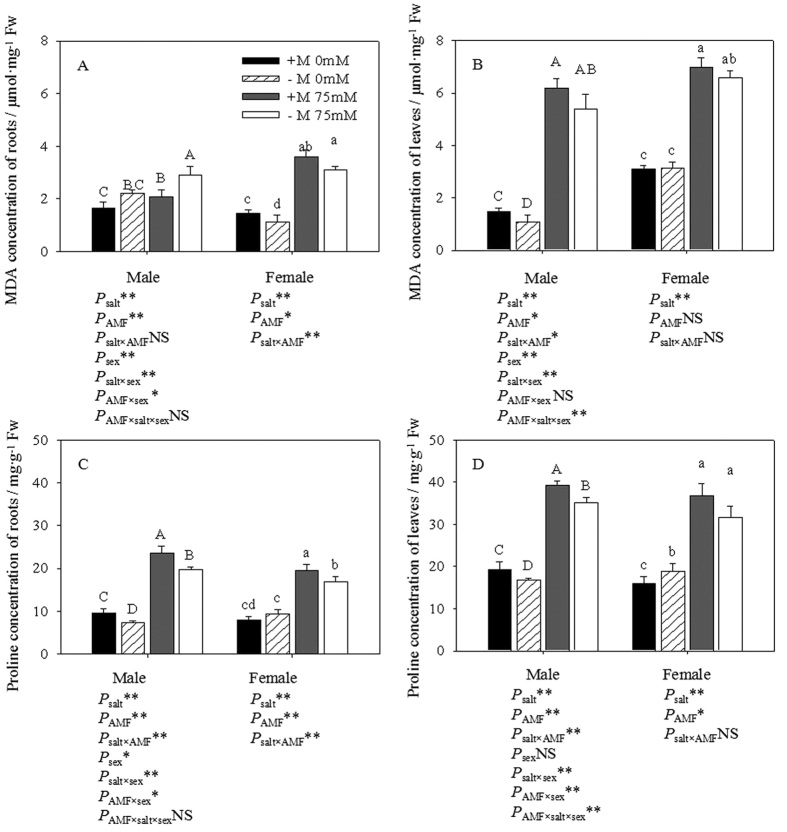
Effects of AMF inoculation on the MDA concentration (**A,B**), proline (**C,D**) and protein content (**E,F**) of leaves and roots under different salt conditions. Abbreviations: MDA: malondialdehyde; +M: AMF inoculation; −M: non-inoculation; 0 mM: without salt stress; 75 mM: under salt stress; AMF: AMF formation. Note: *: significant effect at 0.01 ≤ *P* ≤ 0.05; **: significant effect at *P* ≤ 0.01; NS: no significant effect. Different letters (capital letters for males and lowercases for females) indicate significant difference at p ≤ 0.05.

Salt stress significantly increased the MDA concentration in both the roots and leaves of all individuals (Fig. 5A,B). There was no significant difference in the MDA concentration between males and females in non-salt stress treatments, while females exhibited a higher MDA concentration than males under salt stress. The MDA concentrations in the leaves were 61.55% and 77.42% higher in inoculated and non-inoculated females under salt stress than those not growing under without salt stress, respectively, and 88.19% and 99.41% higher in inoculated and non-inoculated male leaves under salt stress compared with those not growing under saline conditions. Meanwhile, the concentrations in the roots were 96.16% and 106% in females, and 21% and 27.07% higher in males, respectively. Compared with non-inoculated seedlings, the inoculated cuttings grown in the absence of salt stress showed 20.01% higher and 20.25% lower MDA concentrations in the leaves and roots of males, respectively, and 0.78% higher and 7.04% lower concentrations in females. However, when exposed to salt stress, the inoculated male cuttings had 13.22% higher and 27.43% lower MDA concentrations in the leaves and roots, respectively, when compared to non-inoculated seedlings, and the concentrations were 5.71% and 11.66% lower, respectively, in females. Two-way ANOVAs indicated that the MDA concentrations in both the roots and leaves were significantly affected by salt in both sexes and by both salt and AMF in males. Three-way ANOVAs illustrated that gender markedly affected the MDA concentrations and that the MDA concentration in roots was significantly affected by the salt × sex × AMF interaction.

### Proline and protein content measurement

Salt stress significantly increased the proline content in the same way as it affected the MDA concentration (Fig. 5C,D). Differences were observed in the proline content between males and females, where, except for the non-salt and non-inoculated treatments, males exhibited higher proline levels than females irrespective of AMF inoculation under salt stress. Compared with the seedlings that were not exposed to salt stress, the content of proline in the leaves was 100.73% and 71.49% higher in salt-stressed inoculated and non-inoculated females, respectively, and 81.58% and 91.64% higher in males. Meanwhile, the proline content in the roots was 121.08% and 58.53% higher in inoculated and non-inoculated females, respectively, and 120.19% and 126.85% higher in males. When subjected to salt stress, the inoculated male cuttings showed 9.78% and 21.33% higher proline contents in the leaves and roots, respectively, than in un-inoculated seedlings, and the proline contents were 3.22% and 13.43% higher in females. In terms of the protein content, except for the protein content of female roots, there were no significant differences among the treatments (Fig. 5E,F). However, we can see higher protein content in the salt treatments than in the non-salt treatment except in the case of female roots. Two-way ANOVAs indicated that the proline content in both the roots and leaves was significantly affected by salt and AMF inoculation in both sexes. Three-way ANOVAs illustrated that there was a significant difference between genders and that the MDA concentration in leaves was significantly affected by the salt × sex and AMF × sex interactions.

### Reactive oxygen species (ROS)

**Figure 6:**
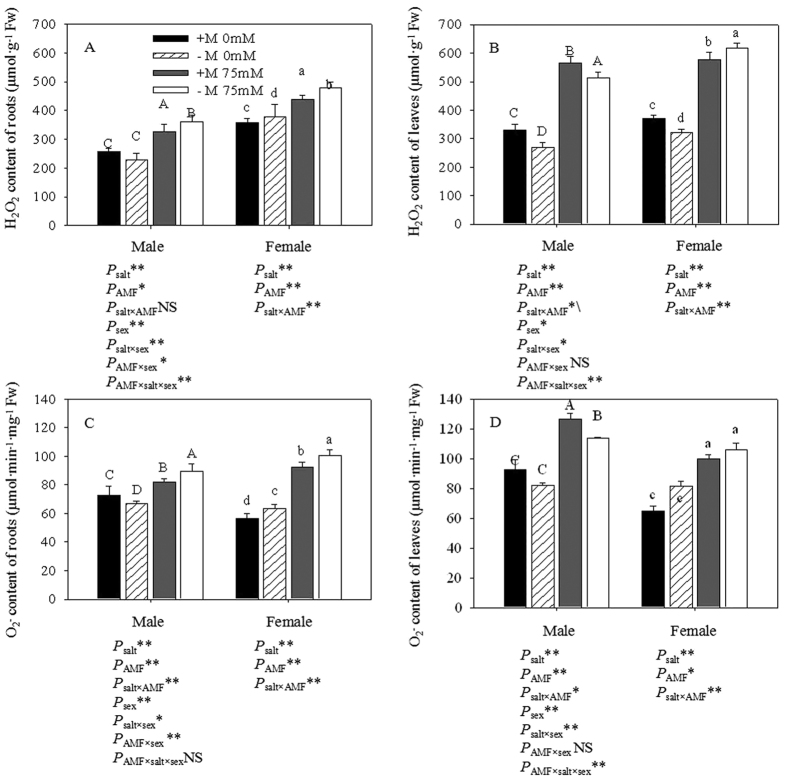
Effects of AMF inoculation on the H_2_O_2_ (**A,B**) and O^2−^ (**C,D**) content of leaves and roots under different salt conditions. Abbreviations: +M: AMF inoculation; −M: non-inoculation; 0 mM: without salt stress; 75 mM: under salt stress; AMF: AMF formation. Note: *: significant effect at 0.01 ≤ *P* ≤ 0.05; **: significant effect at *P* ≤ 0.01; NS: no significant effect. Different letters (capital letters for males and lowercases for females) indicate significant difference at p ≤ 0.05.

Compared with the seedlings that were not exposed to salt stress, the O_2_^−^ generation and H_2_O_2_ content of male and female seedlings subjected to the salt treatment were significantly higher, especially in non-inoculated seedlings (Fig. 6). Under the condition of non-salt stress, inoculated males had 4.13% lower and 6.01% higher H_2_O_2_ contents in the roots and leaves, respectively, compared to non-inoculated seedlings, while these values were 9.11% and 8.84% higher in the roots and leaves of females. Under salt stress conditions, inoculated males had 11.47% lower and 8.87% higher H_2_O_2_ contents in the roots and leaves, respectively, than non-inoculated plants, while these values were 6.63% and 8.39% lower in the roots and leaves of females. The variation in the trends of O_2_^−^ content and H_2_O_2_ content was similar. Under non-salt conditions, the inoculated males had 6.03% and 10.64% higher O_2_^−^ contents in the roots and leaves, respectively, than those that were non-inoculated, and these values were 7.21% and 11.40% lower in the roots and leaves of inoculated females. When exposed to salt stress, the inoculated male cuttings had 10.65% lower and 9.52% higher O_2_^−^ contents in the roots and leaves, respectively, than non-inoculated plants, and these values were 8.66% and 5.70% lower in the roots and leaves of females. Three-way ANOVAs showed that the contents of O_2_^−^ and H_2_O_2_ in the leaves were significantly affected by sex and the interactions of salt × sex and salt × sex × AMF. The rates of O_2_^−^ generation in the roots were significantly affected by sex and the interactions of salt × sex, AMF × sex and salt × sex × AMF, while the H_2_O_2_ content was significantly affected by the interactions of salt × sex and AMF × sex.

### Antioxidant enzymes activity

**Figure 7:**
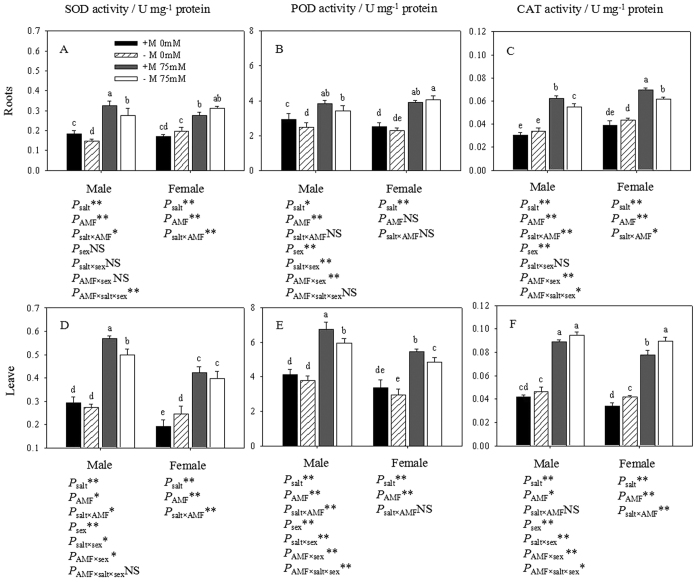
Effects of AMF inoculation on the SOD (**A,D**), POD (**B,E**) and CAT (**C,F**) activity of leaves and roots under different salt conditions. Abbreviations: SOD: superoxide dismutase; POD: peroxidase; CAT: catalase; +M: AMF inoculation; −M: non-inoculation; 0 mM: without salt stress; 75 mM: under salt stress; AMF: AMF formation. Note: *: significant effect at 0.01 ≤ *P* ≤ 0.05; **: significant effect at *P* ≤ 0.01; NS: no significant effect. Different letters (capital letters for males and lowercases for females) indicate significant difference at p ≤ 0.05.

The antioxidant enzyme activities in the two genders showed similar trends as those of the O_2_^−^ and H_2_O_2_ contents. Salt stress activated antioxidant enzymes, including SOD, POD and CAT, in both genders (Fig. 7), and they were measured in the leaf and root tissues of non-inoculated and inoculated cuttings as indicators of oxidative stress caused by salt. Across all enzymes assessed, the activities of antioxidant enzymes in the roots of inoculated males were generally greater than those of non-inoculated cuttings subjected to salt. In the absence of NaCl, no significant differences in antioxidant enzyme activities were detected between the non-mycorrhizal and mycorrhizal roots of females.

The inoculated male cuttings subjected to NaCl showed higher SOD activity (49.30%), POD activity (40.87%), and CAT activity (12.91%) in the male roots than non-inoculated cuttings, and lower SOD activity (11.17%), POD activity (4.38%), and higher CAT activity (13.13%) were found in the roots of inoculated females compared to non-inoculated females. AMF inoculation had a similar impact on the antioxidant activities in the leaves of both genders. The leaves of inoculated males showed 14.44% higher SOD activity, 13.15% higher POD activity, and 5.61% lower CAT activity than non-inoculated leaves, while AMF inoculation resulted in higher SOD activity (6.53%), higher POD activity (11.86%), and lower CAT activity (12.77%) in female leaves under salt stress. In view of gender, the antioxidant activities in the leaves of males were significantly higher than those of females. Three-way ANOVAs showed that the SOD activity in roots was affected by the interaction of AMF × salt × sex, the POD activity in roots was affected by the interaction of salt × sex and AMF × sex, and the CAT activity in roots was affected by the interactions of AMF × sex and AMF × sex × salt. Furthermore, the antioxidant activities in the leaves of both genders were affected by the interactions of salt × sex and AMF × sex.

### The concentration of Na^+^ and Cl^−^

**Figure 8:**
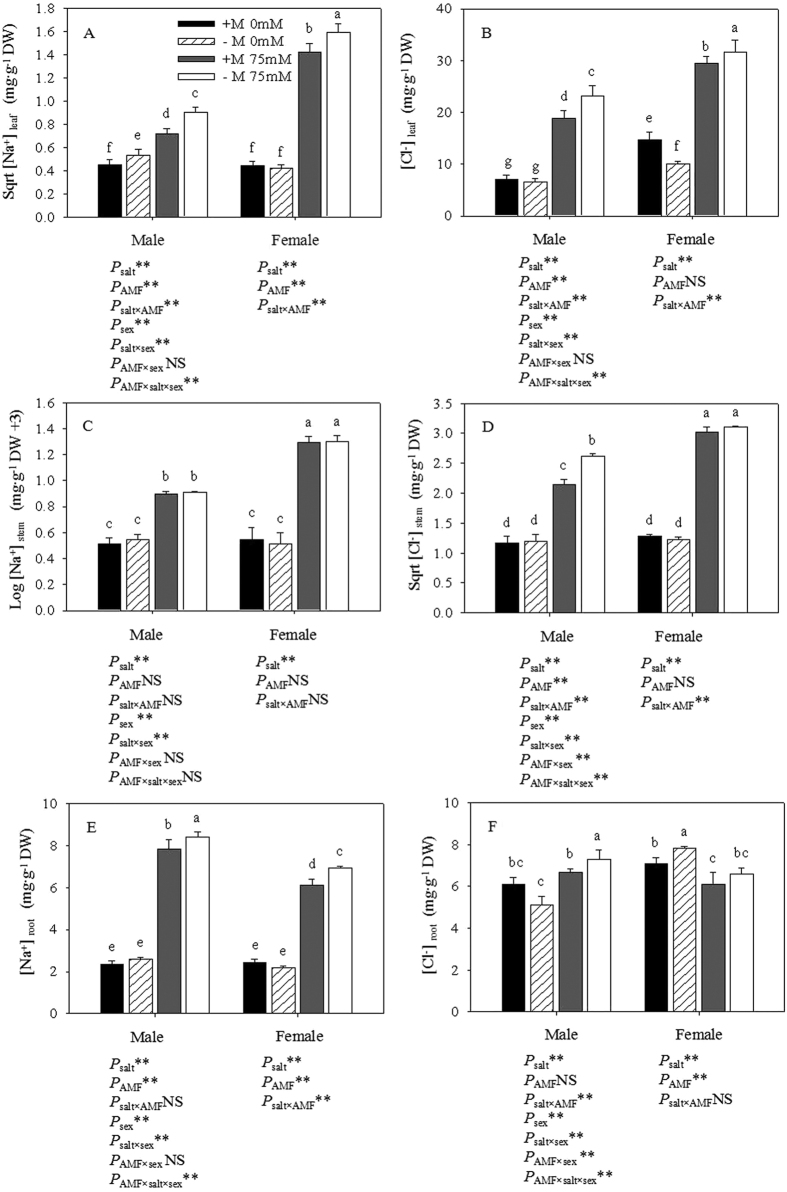
Effects of AMF inoculation on the Na^+^ (**A,C,E**) and Cl^−^ (**B,D,F**) concentrations of leaves, stems and roots under different salt conditions. Abbreviations: +M: AMF inoculation; −M: non-inoculation; 0 mM: without salt stress; 75 mM: under salt stress; AMF: AMF formation. Note: *: significant effect at 0.01 ≤ *P* ≤ 0.05; **: significant effect at *P* ≤ 0.01; NS: no significant effect. Different letters (capital letters for males and lowercases for females) indicate significant difference at p ≤ 0.05.

From our results, we could determine know that the concentrations of Na^+^ and Cl^−^ in the leaves, stems and roots of males were different than those in females under the salt stress treatment (Fig. 8). Except for the Cl^−^ levels in the female roots, salt stress induced the significant accumulation of Na^+^ and Cl^−^ in the leaves, stems and roots of both sexes. The cuttings that had been inoculated with AMF had significantly lower Na^+^ and Cl^−^ contents in the leaves than those of the non-inoculated cuttings under salt stress. Meanwhile, the reductions in the Na^+^ and Cl^−^ levels induced by AMF in males were more obvious than those in females. Compared with the control condition, salt stress induced an increase in the Na^+^ concentration in the leaves of both sexes, especially in females. By contrast, the concentrations of Na^+^ were higher in male roots than in those of females when subjected to salt stress. Na^+^ concentrations in the aboveground parts were considerably lower than in the underground parts. When exposed to salt stress, the accumulation of Cl^−^ significantly increased in the aboveground parts of both genders, and the Cl^−^ levels in the aboveground parts were considerably higher than in the underground parts. Furthermore, the Cl^−^ concentration exhibited a significant increase under salt stress in male roots, while there was no significant difference in female roots. Two-way ANOVAs showed that the Na^+^ and Cl^−^ concentrations in the leaves and the Cl^−^ concentrations in the stems of both genders were significantly affected by the interaction of salt × AMF. The concentrations of Na^+^ in male roots and of Cl^−^ in female roots were not affected by the interaction of salt × AMF. The Cl^−^ concentrations of the leaves, stems and roots of both sexes were all significantly affected by the combined sex × salt interaction (P < 0.05). Only the Cl^−^ concentrations in the stems and roots were significantly affected by the combined sex × AMF interaction. All of the above indexes were significantly affected by AMF.

## Discussion

Salt is one of the detrimental factors in plant growth and development^17^. In this study, we specifically tested the growth parameters, antioxidant defense systems and photosystem II efficiency of females and males subjected to AMF inoculation and salt stress to determine whether there were differences in the mechanisms of the effects of AMF in the two genders under salt stress. The aim of our study was to investigate the effect of *R*. *intraradices* on growth parameters, photochemistry activity, membrane lipid peroxidation and antioxidant enzyme activity under salt stress to further understand salt tolerance mechanisms in dioecious plants inoculated with AMF. In addition, we aimed to discover the potential role of AMF in alleviating gender imbalance. The response to salt involves morphological, physiological and biochemical changes, reflected by limited plant size, damaged cell membranes and stimulated antioxidant responses. Namely, sexual differences were observed in the growth parameters, photochemistry activity and antioxidant responses to salt stress and AMF inoculation in *P. cathayana* seedlings. According to our research, AMF inoculation contributed to the induction of plant salt resistance mainly by enhancing plant growth, chlorophyll fluorescence, antioxidant enzymes activities and the concentrations of antioxidant molecules to protect plant cells from oxidative damage, especially under salt stress, which showed an agreement with previous researches^25^.

*P*. *cathayana* cuttings had a high mycorrhizal dependency under saline conditions, suggesting that poplar is a suitable species for AMF^26^. In addition, in our study, the AMF colonization of *P*. *cathayana* cutting roots was suppressed by salt stress, which was mainly due to the direct effect of salt stress on fungi. Salt stress could hamper the colonization capacity by inhibiting spore germination^27^, hyphal growth in soil^25^ and hyphal spread after initial colonization^9^.

Salt stress is limited not only by the AMF colonization capacity, but also plant growth, which supported previous findings by Greenway and Munns^28^. The early response of plants to salt stress is an avoidance mechanism that functions by regulating the growth rate, visible as a reduction in height (GH), ground diameter (GGD) and leaf area (LA). One of the responsed of plants to salt is a decreased RWC, reflected by a higher osmotic potential arising from salt, which was detected in our study. Salt stress induces a decrease in plant RWC, as reported in wheat^29^ and melon^30^. With a reduction in RWC, a loss of turgor results in a limitation in the water available for cell expansion processes. Our results for the series of plant responses to salt stress were consistent with most studies: the limitation of salt on growth diameters^31^,^32^. However, the responses to salt stress and AMF inoculation differed between males and females: Females showed a higher GH than males without AMF inoculation in the absence of NaCl, while males showed a higher GH than females with AMF inoculation under saline conditions. This suggested that AMF had different impact on the two genders, as described by Lu *et al*.^32^ and Li *et al*.^20^,^21^. We hypothesized that sex-specific differences in life-history traits and their influence on the costs of reproduction could contribute to the variation, as supported by Field *et al*.^33^.

According to our study, salt stress significantly decreased the non-photochemical quenching coefficient (qN), photochemical quenching coefficient (qP), the maximal photosystem II quantum yield (Fv/Fm) and the efficiency of photosystem II in *P. cathayana*. The alteration to photochemistry activity due to NaCl could be attributed to Na^+^ and Cl^−^ toxicity, subsequently followed by oxidative stress. Under saline conditions, males showed a higher maximal photosystem II quantum yield (Fv/Fm) and efficiency of PSII than females, illustrating that females suffered more severely disorder in the electron transport chain of PSII. In addition, the mycorrhizal cuttings exhibited better performance in photosystem II when subjected to salt stress. The higher values of photosynthetic efficiency in the mycorrhizal cuttings indicated that the photosynthetic apparatus of *P. cathayana* was less damaged by the salt stress imposed. Indeed, several studies have shown a positive correlation between the tolerance to salt stress and the maintenance of the efficiency of photosystem II. In addition, better performance of photosystem II in *P. cathayana* cuttings could eventually lead to lower ROS production in both sexes.

Salinity, as one type of environmental stress, increased the MDA concentration in both sexes, especially in non-inoculated seedlings, which supported previous studies by Sudhakar *et al*.^34^. In this study, compared with males, females were more sensitive to salt stress and suffered more oxidative damage, reflected by higher concentrations of MDA. Parallel to our results, previous studies have shown that low MDA content is important in terms of salt tolerance^35^. In addition, peroxidation of membrane lipids has often been used as an indication of membrane damage and leakage under saline conditions^34^. Furthermore, AMF inoculation caused significantly lower MDA concentrations in the roots of both sexes, therefore, the trends in the leaf concentrations of MDA in males and females were the same. Our results suggested that inoculated seedlings have better salt tolerance than non-inoculated seedlings.

Generally, proline has a key role in the stabilization of cellular protein and membranes under high salt conditions, and the accumulation of proline has been observed in many plants under salt stress^36^. Proline can act as an indicator of salt-stress injury as well as an osmo-protectant. Salt stress induced proline accumulation^37^.Several authors have reported that mycorrhizal plants subjected to various salt levels accumulate less proline than non-mycorrhizal plants^19^,^31^. However, parallel to our results, a higher proline content was observed in mycorrhizal plants than in non-mycorrhizal plants under saline conditions^38^,^39^, suggesting that proline accumulation might be necessarily for AMF colonization as well as a response strategy in some plants. Besides, males and females showed different responses to salt and AMF inoculation in terms of the proline content in both roots and leaves, and such differences were also detected in other parameters. The different levels of tolerance and resistance to stress shown by male and female poplars were considered to reflect a cascade of complex mechanisms^40^.

In general, salt stress can cause the excessive accumulation of reactive oxygen species (ROS) and ROS scavenging within plants^41^. The generation of excess ROS can result in an imbalance of O_2_^−^ and H_2_O_2_, which are generally maintained in a dynamic balance, and subsequently to oxidative damage to many bio-molecules, as demonstrated by cellular membranes and proteins^42^. Keeping with this, our study showed that salt stress caused a significant improvement in O_2_^−^ and H_2_O_2_. Similar sex-specific differences in ROS metabolism were also observed by Han *et al*.^3^. With regard to the effect of gender, males appeared to adopt an avoidance strategy by restricting the access of toxic ions to sensitive organs such as leaves, to lessen their damage to plant physiology. These results showed that females were more sensitive to stress conditions than males^4^,^43^. H_2_O_2_ has been reported to accumulate in clumped or less branched arbuscules and intercellular fungal hyphae in AMF-colonized roots^44^. In our study, salt stress triggered a notable increase in O_2_^−^ and H_2_O_2_ contents, and AM symbiosis mediated the different extents of ROS accumulation in the different organs within plants. It has been documented that H_2_O_2_ can be specifically transported through aquaporin channels^45^. As a result, mycorrhizal hyphae might provide a pathway for H_2_O_2_ effluxes from the root to the rhizosphere. Further studies are needed to clarify the function of AMF in the modulation of H_2_O_2_ and O_2_^−^ effluxes. As is well known, the components of ROS can act as signal molecules in defense responses and other physiological processes^46^. However, it was not possible in the present study to determine whether the higher contents of H_2_O_2_ and O_2_^−^ in inoculated plants under salt stress could activate H_2_O_2_ signaling in roots.

Previous studies have shown that the tolerance of plants to salt stress is associated with the induction of antioxidant enzymes and the reduction of oxidative damage. AMF enhances the activity of antioxidant enzymes, which helps plants to cope with the ROS generated by salt stress^18^. To obtain a good understanding of the extent of cell resistance against the oxidative damage induced by salt stress, we measured the activity of the ROS scavenging enzymes SOD, POD and CAT. These antioxidant enzymes not only act as indicators of increasing ROS production but are also the manifestation of antioxidant defense^15^. According to our research, the increase in ROS as signal molecules also induced the expression of antioxidant systems or increased the activity of these enzymes. The activities of SOD (a scavenger of O_2_^−^) and CAT (a scavenger of H_2_O_2_) within plants were significantly higher in AMF than non-AMF plants under both salt conditions, except for a similar leaf CAT activity in AMF and non-AMF plants in the presence of salt ions. These results illustrated that the presence of AMF can induce higher antioxidant enzyme activities as a protective mechanism to prevent the over-accumulation of ROS. This is in agreement with the observations of Bompadre *et al*.^47^ and Zou *et al*.^48^. Based on the protein content results in this study, we suggested that ROS rather than protein content should be used for the correct interpretation of the specific activities of SOD, POD and CAT in leaves and roots. In fact, the detoxification of ROS also involves some other antioxidant enzymes (e.g., ascorbate peroxidase, glutathione reductase, and guaiacol peroxidase) and antioxidants (e.g., ascorbate, glutathione, and tocopherols)^49^, which need to be further studied using mycorrhizal plants under saline conditions.

Most plants were sensitive to high Na^+^ and Cl^−^ concentration. The primary impact of high ionic concentrations was the disturbance of intracellular homeostasis, which leads to the attenuation of metabolic activity and membrane dysfunction, followed by secondary impacts that can lead to cell death^30^. As has been documented extensively, it is essential for plants to maintain low concentrations of toxic ions in leaves to grow and survive under salt stress. It has been found that many plants, e.g., *Populus euphratica*^50^, *Cicer arietinum* L^51^ and *Jatropha curcas*^52^, can effectively prevent Na^+^ and Cl^−^ uptake under salt stress. In our study, under saline conditions, the Na^+^ concentrations in the shoots of male plants were significantly lower than those in the roots, indicating the efficient inhibition of root-shoot Na^+^ transport in males. However, the variation in the Cl^−^ concentration differed from that of the Na^+^ concentration. Thus, we could attribute the cause of these contrasting patterns to the different methods of uptake. Na^+^ enters a plant cell through non-selective cation channels – HKT1 in some species, such as *Solanum lycopersicum*^53^. The lower transportation rate of Na^+^ and Cl^−^ from the root to shoot and compartmentalization endow plants with anti-salt toxicity abilities. Based on the results, we suggested that males have a higher capacity for transporting than females, conferring a higher salt tolerance in males than females. Apart from plants’ internal mechanisms for salt tolerance, AMF can also provide external assistance. Previous studies have reported that mycorrhizal colonization can prevent the disruption of various enzymatic processes by means of preventing Na^+^ translocation to shoot tissue^25^. Our results also showed that the concentration of Na^+^ in mycorrhizal plants was significantly lower than that in non-mycorrhizal plants. Mycorrhizal colonization might also act as a first barrier for ion selection during the fungal uptake of nutrients from the soil or during transfer to the plant host. AMF induced a buffering effect on the uptake of Na^+^ when the content of Na^+^ was within the permissible limit. Indeed, the regulation mechanism involved in ion homeostasis has been supported from both physiological and molecular perspectives in studies of salt stress alleviation by AMF^17^. We hypothesized that AMF inoculation had advantages in both sexes, enabling Na^+^ and Cl^−^ in the leaves and roots to remain low, thereby allowing the seedlings to grow better under salt stress. Hence, we suggested that AMF inoculation alleviated the negative effects of salt by storing Na^+^ and Cl^−^.

## Conclusion

Taken together, salt stress significantly limited the growth of shoots and roots in male and female *P. Cathayana*, and AMF alleviated this negative damage. The symbiosis with *R*. *intraradices* facilitated photochemical activity as well as the pro/antioxidant balance of the host plants under salt stress. Based on our results and the existing literature, mycorrhizal plants are more efficient in oxidative adjustment compared to their non-mycorrhizal equivalents and AMF playe a more important role in roots. Therefore, compared with females, AMF had great advantages in facilitating salt-induced protection for males, including having positive effects on the physiological function and development of leaves and roots, which would be a potentially beneficial means of species continuation.

## Methods

### Plant and soil treatment

The experimental cuttings of *P*. *cathayana* were kept 18 cm in length and 1.2 cm in diameter and were collected from 60 male and 60 female from different trees (120 genotypes) sampled in 15 populations in Sining, Qinghai Province, China. Then disinfected the cuttings with 0.05% KMnO_4_ for 12 h, and rinsed 3 times with sterile deionized water.

Topsoil (0~20 cm) was collected from a poplar nursery in Yangling, Shaanxi province, China. After sieving with a 2 mm sieve, the soil was mixed with fine sand (V/V = 1:1) and autoclaved at 0.11 MPa, at 121 °C for 2 h for use as a soil substrate. The physicochemical properties of the soil substrate were pH 7.6 (soil and water ratio was 1:5), available N 37.31 mg/kg, available P 12.30 mg/kg, available K 132.21 g/kg, and soil organic matter (SOM) 18.74 g/kg.

### AM inocula

The inocula of the AMF *Rhizophagus intraradices* JJ291 (BEG accession 158 at the International Bank for the Glomeromycota; www.kent.ac.uk/bio/beg/) consisted of spores (the spore density was approximately 50 per gram (g) of inoculant), mycelia, root fragments and soil in our study. At the beginning of the experiment, 10 g of inocula was placed in the vicinity of the cuttings in the inoculated treatments, and the other cuttings were inoculated with 10 g of autoclaved inocula with 10 ml of inocula washing solution from live inocula that had been filtered through a 1 μm nylon mesh as controls.

### Plant material and experimental design

The experiment consisted of a randomized complete block design with three factors: sex (male or female), inoculation status (inoculated or not inoculated with *R. intraradices*) and salt (salt or non-salt). There were 15 replicates of each treatment, totaling 120 pots (one plant per pot), such that 30 of each sex were grown under non-saline conditions throughout the entire experiment, while the other 30 pots were subjected to 75 mM of NaCl. Sixty cuttings of each sex were planted in 4.5 l plastic pots filled with 4 kg of homogenized matrix and then grown under semi-controlled environmental conditions in a well-ventilated greenhouse with temperature range of 25~30 °C, a relative humidity range of 60–80% and 12 h of light per day. Thirty plants of each sex were inoculated with AMF inocula (20 g/pot), and the other plants were inoculated with 20 g of autoclaved inocula with 10 ml of inocula washing solution from live inocula filtered through a 1 μm nylon mesh as controls. After the plants had grown for a month, each cutting was supplied with 200 ml Hoagland’s solution every week. The formula for Hoagland’s nutrient solution was modified by Johnson *et al*.^54^. After culturing for 50 days, the cuttings were divided into 2 groups of 15 individuals. One group was provided with 15 mM salt every 2 days for a total of 5 times totally, and the plants were eventually harvested after 1 month.

### AM colonization and mycorrhizal dependency

Mycorrhizal colonization was estimated by the method of Phillips and Hayman^55^. Randomly sampled roots were cleared with running tap water immediately after harvest. The roots were then fixed by FAA, cleared with 10% KOH and stained with 0.05% trypan blue in lacto glycerol. 150 root segments (1 cm long) from each treatment were mounted under a compound light microscope. The colonization of roots by AMF was measured by the method of gridline intersection as described by Giovannetti and Mosse^56^. The data were given as the percentage of root length colonized. At the end of the experiment, all the cuttings were harvested and dried at 70 °C for 48 h to a constant weight and then weighed. The mycorrhizal dependence (MD) was calculated using the following formula^57^:


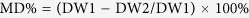


DW1, the dry weight of mycorrhizal plants;

DW2, the dry weight of non-mycorrhizal plants.

### Growth measurement

In order to record the growth responses, the stem length and above-ground diameter of 120 cuttings were measured at the beginning and end of the salt treatments. And the growth rate in terms of height and the stem base diameter divided by days of treatment were calculated. The chlorophyll content was measured at the end of the experiment with a chlorophyll meter (SPAD-502 Plus, Konica-Minolta Holdings, Inc., Osaka, Japan). The leaf area (LA) was determined using coordinatepaper.

### Relative water content (RWC) of leaves determination

Six the fourth or fifth fully expanded leaves from the apex (Leaf Plastochron Index 4–5) from each treatment and sex were collected from six randomly selected seedlings, and weighted (FW), and then kept in distilled water for 24 h, recorded for turgid weight (TW). Then the leaves were dried in hot air oven at 70  °C till constant weight (DW). RWC was calculated as described by Whetherley^58^: RWC (%) = (FW−DW) × 100/ (TW−DW).

### Chlorophyll fluorescence measurement

The fourth and fifth fully expanded leaves were selected for chlorophyll fluorescence measurements using a PAM chlorophyll fluorometer (MINI-Imaging-PAM, Walz, Germany). First, the minimum fluorescence (Fo) and the maximal fluorescence (Fm) were measured after darkening the leaves for 30 min at room temperature. The maximum quantum yield of PSII was calculated by Fv/Fm (Fv = Fm − Fo), while the actual quantum yield of PSII was calculated by ΦPSII = (Fm’ − F)/Fm’. And in light adapted state, the non-photochemical quenching (qN) was calculated by qN = 1− (Fm’ − Fo’)/(Fm − Fo) and the photochemical quenching (qP) was calculated by qP = (Fm’ − F)/(Fm’ − Fo’).

### Antioxidant enzyme extractions and assays

For SOD extraction, samples (0.5 g fresh leaves or roots) were homogenized in a cold medium containing: 50 mM K-phosphate buffer (pH 7.0), 1 mM EDTA, 0.05% (v/v) Triton X-100, 1 mM Na-ascorbate, 1 mM DTT, 0.3 mM PMSF and 10% (w/w) polyvinylpyrrolidone (PVP). After centrifugation at 12,000 × *g*, the supernatant was dialyzed overnight at 4 °C. For POD and CAT extraction, samples (0.5 g fresh leaves or roots) were ground in liquid nitrogen with a pre-cooled mortar and pestle. Subsequently homogenized in a medium with 10% (w/w) polyvinylpolypyrrolidone (PVPP) contained: 0.1 M Tris buffer (pH 8.0), 10 mM DTT, 10 mM MgCl_2_, 50 mM KCl, 1 mM EDTA, 0.1% (v/v) Triton X-100 and 50 μg ml^−1^ phenylmethylsulphonyl fluoride (PMSF).

The antioxidant enzymes were assayed according to Mallick and Mohn^59^. For the SOD assay, the reaction medium contained: 50 mM K-phosphate buffer (pH 7.8), 100 μm EDTA, 13 mM methionine, 75 μm nitroblue tetrazolium (NBT), 2 μM riboflavin and 50 μl enzyme extract. Through measuring its ability to inhibit photochemical reduction of NBT, SOD activity was assayed. The absorbance was measured spectrophotometrically at 560 nm. For POD assay, the reaction mixture contained: 50 mM*p*-chloromercuribenzoate (pCMB) and 0.1% (w/v) dianisidine in methanol: dioxane (1:1 v/v). The absorbance was measured spectrophotometrically at 460 nm. For CAT assay, the reaction was initiated by adding 10 μl of the root and leaf extract to solution. And the reaction mixture contained 50 mM K-phsphate buffer (pH 7.0), among which 10.6 mM H_2_O_2_. Eventually the absorbance was measured spectrophotometrically at 240 nm.

### Lipid peroxidation

Lipid peroxidation levels in leaves and roots of males and females, measured as the concentration of MDA. The concentration of MDA was determined as described by Kramer *et al*.^60^. Fresh leaves and roots were homogenizing (0.5 g) with 10 ml of 10% trichloroaceticacid (TCA), and centrifuged it at 12,000 × *g* for 10 min. Then, adding 2 ml of 0.6% thiobarbituric acid (TBA) into aliquot of 2 ml of supernatant. The mixture was heated in boiling water for 30 min, and then quickly cooled in an ice bath. Measuring the content of MDA by a spectrophotometer (UV-2550, Shimadzu Co. Ltd., Japan) to determine the absorbance of the supernatant at 450, 532 and 600 nm. The formula C (μM) = 6.45 (OD_532_–32s_600_) spectrop_450_ was used to calculate the MDA concentration.

### Proline content and protein concentration determination

The proline content was determined according to Bates *et al*.^61^. Frozen leaf or root tissue (0.5 g) was homogenized with 10 ml of 3% sulfosalicyclic acid at 4 °C and centrifuged at 10,000 × *g* for 5 minutes. The extract was filtered through the Whatman No. 2 filter paper. In a test tube, 2 ml of the filtrate, 2 ml of acid-ninhydrin and 2 ml of pure acetic acid were mixed, and incubated at 100 °C for 1 h. The reaction eventually terminated in an ice bath and then 4 ml of toluene was added and the mixture was shaken well for 20 s. The upper layer was collected and the absorbance was measured spectrophotometrically at 520 nm. Proline concentration was determined based on a standard curve (range 0–50 mg ml^−1^). The protein concentration of roots and leaves was determined based on the Bradford method^62^.

### Quantification of reactive oxygen species (ROS)

The O_2_^−^ and H_2_O_2_ contents were measured according to Apel and Hirt^63^. For the determination of O_2_^−^, fresh leaves and roots (0.5 g) were homogenized with 1 ml hydroxylamine hydrochloride for 1 h. Then adding 1 ml of p-aminobenzene sulfonic acid and 1 ml of α-naphthylamine into above test tube and keeping at 25 °C for 20 min. The absorbance was measured spectrophotometrically at 530 nm by the standard curve of NaNO_2_. For the determination of H_2_O_2_, fresh leaf and root tissue were homogenized with titanium tetrachloride, eventually forming a H_2_O_2_ titanium complex. Analogously, the content of H_2_O_2_ was measured at 415 nm by the standard curve of known H_2_O_2_ concentration.

### Na^+^ and Cl^−^ determination

Leaves, stems and roots of males and females were groud to homogeneous powder and passed through a 20 μm mesh screen after being dried at 80 °C until constant weight. The Na^+^ concentration was determined according to Peng *et al*.^64^. Dry powders of leaf, stem and root tissue were extracted with HCl overnight at 37 °C. After centrifugation at 10,000 × *g* for 10 min, the supernatants were diluted and determined by an atomic absorption spectrophotometer (Z-2000, Shimadzu, Japan). The Cl^−^ concentration was measured by the method of Chen *et al*.^65^. An aqueous extract of the dry powder was mixed with K_2_Cr_2_O_4_ solution used as color indicator for isoionic point determination. An adequate AgNO_3_ solution was served to precipitate and measure it.

### Statistical analysis

Two-way and three-way of variance (ANOVA) were used to test experimental data by the statistical software package SPSS 17.0 (SPSS Inc., IL, USA). All values are means of six replicates. Significant differences were calculated by Duncan’s multiple-range tests (P < 0.05). Two-way ANOVAs were conducted to determine the significance of salt stress, AMF inoculation and their interaction in each sex. Three-way ANOVAs were conducted to determine the significance of sex, salt × sex, AMF inoculation × sex, and sex × salt × AMF inoculation. Data were transformed prior to ANOVA analysis with using a log transformation (Na^+^ concentration in stem tissue) and square root transformation (Na^+^ concentration in leaf tissue and Cl^−^ concentration in stem) to obtain a normal distribution and homogeneity of variances^66^.

## Additional Information

**How to cite this article**: Wu, N. *et al*. Comparative photochemistry activity and antioxidant responses in male and female *Populus cathayana* cuttings inoculated with arbuscular mycorrhizal fungi under salt. *Sci. Rep*. **6**, 37663; doi: 10.1038/srep37663 (2016).

**Publisher's note:** Springer Nature remains neutral with regard to jurisdictional claims in published maps and institutional affiliations.

## Figures and Tables

**Table 1:** Total colonization rates of inoculated *P*. *cathayana* males and females under different salt conditions.

Treatments	Male		Female	
	0 mM	75 mM	0 mM	75 mM
Colonization rate (%)	89.09 ± 3.92	88.03 ± 4.32	88.11 ± 3.01	90.94 ± 4.27

Note: The data are means ± SD (n = 6). Different lowercase letters (a, b) indicate a significant difference at *P* ≤ 0.05.
